# Sex differences in vascular function among individuals with heart failure: a systematic review

**DOI:** 10.1016/j.ijcha.2026.101979

**Published:** 2026-07-28

**Authors:** Sara Younas, Nduka C. Okwose, Amy S. Fuller, Renae J. Stefanetti, Sarah J. Charman, Fatima Bano, Milan Dobric, Milovan Bojic, Petar M. Seferovic, Prithwish Banerjee, Djordje G. Jakovljevic

**Affiliations:** a Clinical Sciences and Translational Medicine, Research Centre for Discoveries in Life Sciences, Coventry University, Coventry, UK; b Institute for Cardio-Metabolic Medicine, University Hospitals Coventry and Warwickshire NHS Trust, Coventry, UK; c Translational and Clinical Research Institute, Faculty of Medical Sciences, Newcastle University, Newcastle upon Tyne, UK; d Newcastle upon Tyne Hospitals NHS Foundation Trust, Newcastle upon Tyne, UK; e Institute for Cardiovascular Diseases Dedinje, Serbia; f Faculty of Medicine, University of Belgrade, and Serbian Academy of Sciences and Arts, Belgrade, Serbia; g Clinical Trials Unit, Warwick Medical School, University of Warwick, Coventry, UK

**Keywords:** HF, HFpEF, Vascular function, Arterial stiffness, Sex differences

## Abstract

Heart failure with preserved ejection fraction (HFpEF) is more prevalent in women, whereas heart failure with reduced ejection fraction (HFrEF) predominates in men. Despite these well-established epidemiological differences, sex-specific alterations in vascular function in heart failure (HF) remain poorly characterised. This systematic review, using a narrative synthesis approach, evaluated sex-related differences in vascular function in individuals with HF. The review was prospectively registered with PROSPERO (CRD42024617745). MEDLINE and CINAHL were searched from inception to 26th November 2024 for studies reporting sex-stratified measures of arterial stiffness among individuals with HF. Nine studies met the eligibility criteria for inclusion (*n* = 2820; men: *n* = 1390, women: *n* = 1430). Of the included studies, 78% were characterised as HFpEF. Compared with men, women exhibited a higher pulsatile arterial load and lower arterial compliance. Representative findings from individual studies showed that women exhibited a higher augmentation index (28.9 ± 13.7% vs 21.7 ± 11.9%, *p* < 0.001) and augmentation pressure (19.1 ± 12.4 vs 13.7 ± 10.1 mmHg, *p* = 0.003). Body mass index (BMI) showed variable relationships with arterial stiffness indices, including positive associations with pulse wave velocity (*r* = 0.24, *p* < 0.01), central pulse pressure (*r* = 0.33, *p* < 0.001), and augmentation index (*r* = 0.23, *p* = 0.01), but an inverse relationship was found with cardio-ankle vascular index (*r* = −0.204, p < 0.001). Importantly, sex differences in HFpEF remained significant after adjustment for BMI. Women with HF exhibit higher pulsatile arterial load and reduced arterial compliance compared with men, which remain after adjustment for BMI. Sex-specific vascular dysfunction contributes to HF pathophysiology and supports the need for sex-informed assessment.

## 1 Introduction

Heart failure (HF) is a clinical syndrome characterised by symptoms and signs resulting from structural or functional cardiac abnormalities [1]. Diagnosis of HF is detected by elevated natriuretic peptide levels and/or objective evidence of pulmonary or systemic congestion [1]. HF is recognised as a global health concern, affecting approximately 64 million individuals worldwide and placing a significant financial burden on healthcare systems [2].

**Fig. 1:**
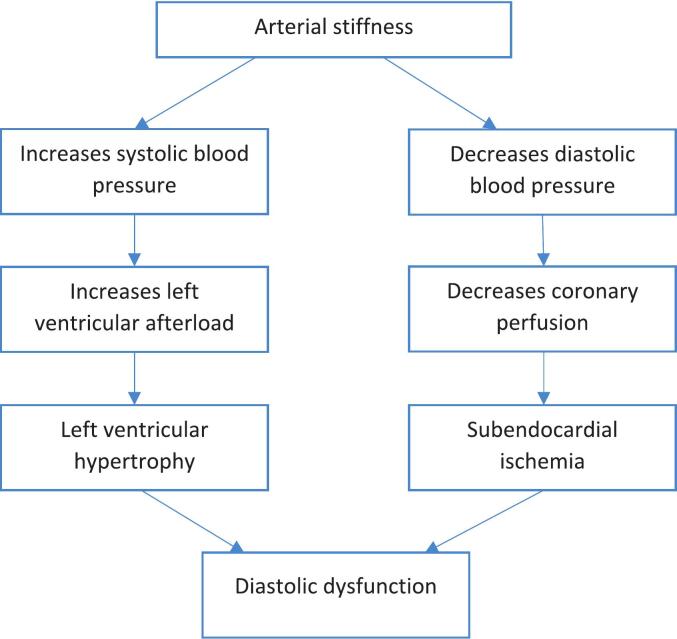
**Pathophysiological pathway of relationship between increased arterial stiffness and diastolic dysfunction**.

Arterial stiffness, a marker of vascular ageing, defined as the reduced capacity of arteries to efficiently expand and contract in response to variations in blood pressure (BP), contributes significantly to left ventricular (LV) afterload and diastolic dysfunction in HF [3]. To sustain continuous blood circulation, the elasticity of healthy arteries allows them to expand and accommodate the surge of blood flow during systole and recoil during diastole [4]. Arterial stiffening is implicated in the aetiology of pathophysiological changes associated with ageing and the development of medical conditions including atherosclerosis, hypertension, and chronic inflammation. This loss of elasticity is primarily due to structural changes in the arterial walls, such as increased collagen and reduced elastin content [5]. Consequently, the reduced ability of the arteries to accommodate pulsatile blood flow leads to higher systolic BP and lower diastolic BP [6]. The strain on the heart muscle as a result of reduced arterial compliance eventually results in an increase in cardiac workload and detrimental effects on the structure and function of the heart (Fig. 1) [7].

HF phenotypes vary between men and women [8]. Women are more likely to be diagnosed with HF with preserved ejection fraction (HFpEF), whereas men more commonly present with HF with reduced ejection fraction (HFrEF) [9], a pattern partly attributed to the cardio protective effects of estrogen [10]. Women are also typically diagnosed with HF at an older age than men, which may further contribute to the higher prevalence of HFpEF in women [11]. It has also been suggested that a correlation between LV diastolic function and arterial stiffness may be a significant factor causing this sex difference [3]. These observations highlight the need to explore vascular mechanisms, particularly arterial stiffness, as a potential contributor to sex-specific HF phenotypes.

No comprehensive synthesis currently exists on sex-related differences in arterial stiffness among individuals with HF. Previous reviews to date have focused on general HF pathophysiology or treatment strategies but have not systematically examined arterial stiffness by sex [12], [13]. The purpose of this systematic review was to examine sex-related differences in vascular function in individuals with HF.

## 2 Methodology

### 2.1 Registration

This systematic literature review was prospectively registered in PROSPERO (CRD42024617745).

### 2.2 Search strategy

A comprehensive search was conducted in MEDLINE and the Cumulative Index to Nursing and Allied Health Literature (CINAHL) databases using the following keywords and Boolean operators.

1. Heart Failure and Related Terms:

Heart failure OR hf OR cardiac failure OR chf OR cardiac overload OR chronic heart failure.

OR congestive heart failure OR ccf OR cardiac dysfunction OR ventricular dysfunction OR.

Systolic dysfunction OR myocardial dysfunction OR cardiac insufficiency OR heart.

Insufficiency OR LVSD OR diastolic dysfunction.

2. Vascular Function and Related Terms:

Arterial stiffness OR pulse wave analysis OR vascular stiffness OR aortic stiffness OR.

Arterial rigidity OR vascular rigidity.

Search filters were adjusted to ensure that studies including both men and women were eligible for inclusion. Searches covered all years from database inception to 26th November 2024. Grey literature databases such as conference proceedings and editorials were searched for relevant literature and reference lists of all included studies were screened for potentially eligible articles. Studies published in English, or non-English studies with available translations were included. Non-English publications without translations were excluded due to feasibility limitations.

### 2.3 Inclusion criteria

Studies were eligible for inclusion if they included: (i) adults (≥18 years) with a clinical diagnosis of HF (any phenotype); (ii) reported sex-specific analysis; (iii) assessed arterial stiffness; (iv) published in English or had an English translation available; (v) used an observational study design (cross-sectional, cohort, case-control), a randomised controlled trial, or a systematic review with quantitative or mixed-methods data.

### 2.4 Exclusion criteria

Studies were excluded if they: (i) involved paediatric populations; (ii) did not report arterial stiffness; (iii) did not report sex-specific data, as these were essential to address the primary objective of evaluating sex-related differences and would not allow meaningful comparison between men and women; or (iv) were case reports, editorials, letters, conference abstracts, or non-systematic narrative reviews that were unrelated to HF or lacked sex-specific data.

### 2.5 Study selection and data extraction

**Fig. 2:**
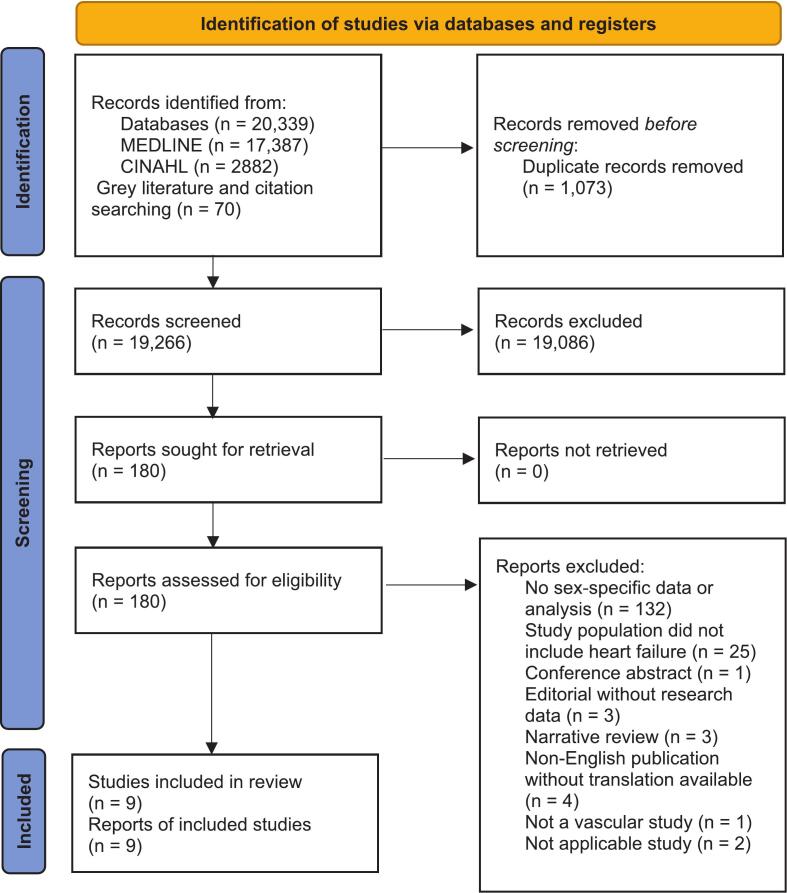
PRISMA 2020 flow diagram outlining the study selection process.

All articles retrieved through database searches were uploaded in Rayyan software [14], and duplicates were removed. Three reviewers independently screened titles and abstracts in accordance with the predefined eligibility criteria, followed by full-text assessment of all studies deemed potentially eligible. Any discrepancies between the reviewers were resolved through discussion or by consulting a fourth reviewer. The study selection process was documented using Preferred Reporting Items for Systematic Reviews and Meta-Analyses (PRISMA 2020) flow diagram (Fig. 2) [15].

Data from included studies were independently extracted by four reviewers using a predefined data extraction template in Microsoft excel. Extracted data included study characteristics (author, year, country, and study design), sample size, HF phenotype, methods for measurement of arterial stiffness and sex-stratified outcomes. Any discrepancies were resolved through group discussion. The methodological quality of the included studies was assessed using the Cochrane Risk of Bias tool for randomised trials [16] and an adapted version of the checklist for observational studies described in Appendix 5 of Brazzelli et al. [17]. Assessments were conducted by one reviewer and independently checked for accuracy by a second reviewer, with any discrepancies resolved through discussion.

### 2.6 Data synthesis

Studies were grouped according to (1) the type of heart failure (HFpEF, HFrEF, and mixed HF), (2) the technique used to assess arterial stiffness including carotid femoral pulse wave velocity (cfPWV), augmentation index (AIx), central blood pressure indices, the cardio-ankle vascular index (CAVI), and impedance-derived measures such as aortic characteristic impedance (Zc) and proximal arterial compliance (PAC), (3) sex-specific findings including age, BMI, and comorbidities were extracted where reported, and (4) study design (cross-sectional, prospective cohort, and post-hoc analyses of randomised trials).

Descriptive statistics are reported as means ± standard deviations (SD) for continuous variables and counts with percentages for categorical variables, as originally presented in the included studies. No new statistical calculations were performed. Reported numerical values are derived from individual studies for illustrative purposes and do not represent pooled or aggregated estimates across studies.

## 3 Results

### 3.1 Study selection

The database search identified 20,339 records. After removal of duplicates, 19,266 studies were screened by title and abstract, of which 19,086 were excluded. A total of 180 studies were eligible for full-text screening, resulting in the exclusion of 171 studies. Finally, nine studies met the inclusion criteria, and were included in the narrative synthesis (Fig. 2).

### 3.2 Study characteristics

A total of 2820 patients were included across the nine studies, including 1390 men and 1430 women. Sample sizes ranged from 58 to 767 participants. Of the included studies, HFpEF was the predominant phenotype (*n* = 7) [18], [19], [20], [21], [22], [23], [24], one study evaluated mixed phenotype HF [25], and one assessed HFrEF [26]. A summary of the characteristics of included studies is demonstrated in supplemental material Table 1.

### 3.3 Methodological quality assessment

The included randomised controlled trial deemed a low risk of bias, with adequate randomisation, allocation concealment, blinding, and complete outcome data (Supplementary Table 2.2) [19]. Among the eight non-randomised studies, the risk of bias varied, with four studies classified as low risk, three as moderate risk, and one as high risk, indicating a heterogeneous methodological quality across observational designs [18], [20], [21], [22], [23], [24], [25], [26].

Most studies clearly described inclusion/exclusion criteria and reported outcomes reliably. Reporting of missing data and withdrawals was inconsistent, with several studies providing no information on the handling of missing data. Most studies reported prespecified outcomes and disclosed funding or conflicts of interest, although the level of transparency varied. Detailed domain based risk of bias assessments for each study are presented in Supplementary Table 2.1. Information regarding prospective registration or the availability of study protocols was not consistently reported across the included studies.

### 3.4 Sex-related differences in arterial stiffness

**Fig. 3:**
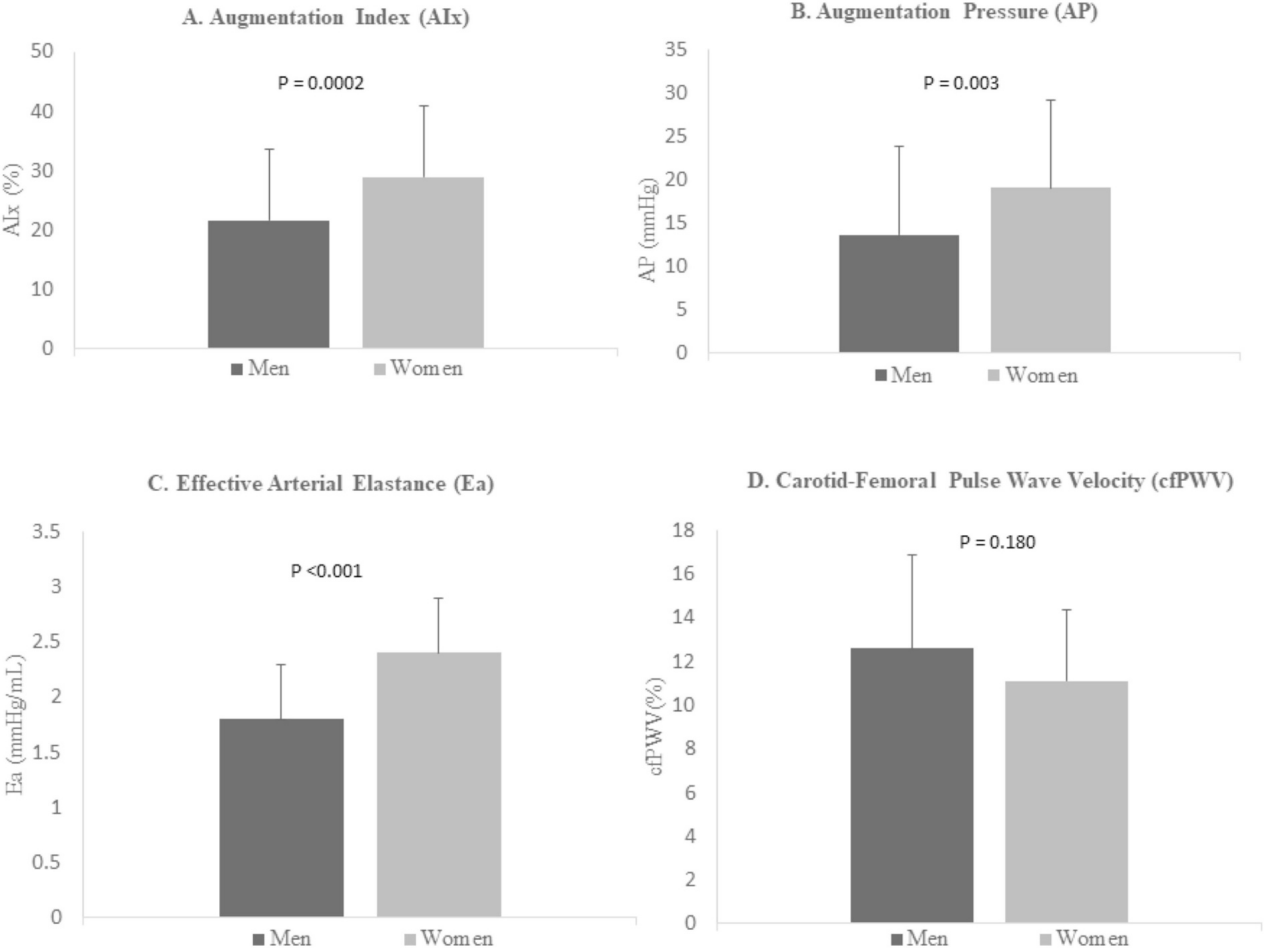
Sex differences in arterial stiffness indices from representative studies. Panel A: Women showed higher AIx compared with men. Panel B: AP was higher in women. Panel C: Ea was higher in women. Panel D: cfPWV was slightly lower in women than men; error bars represent SD for AIx, AP, and cfPWV, and IQR for Ea. Values are derived from individual studies and are presented for illustrative purposes only; no meta-analysis or pooled estimates were performed. AIx = Augmentation Index; AP = Augmentation Pressure; cfPWV = Carotid–Femoral Pulse Wave Velocity; Ea = Effective Arterial Elastance.

Eight of the nine included studies reported sex-specific analyses of arterial stiffness in individuals with HF [18], [19], [20], [21], [22], [23], [24], [26]. Five studies assessed AIx, of which all consistently found higher AIx values in women compared to men [18], [22], [23], [24], [26], indicating greater wave reflection and pulsatile load in women. A visual summary of sex differences in AIx, AP, and Ea is presented in Fig. 3.

In contrast, three studies evaluated cfPWV [22], [23], [26], among which two studies reported no significant sex differences [23], [26], while one study observed slightly lower cfPWV in women as compared to men (*p* = 0.015) [22]. Table 3 presents the detailed sex-stratified results.

Body mass index (BMI) was reported in five studies [18], [19], [22], [25], [26] and was generally higher in women than men (e.g., Crosier et al.: 34.9 vs 31.5 kg/m^2^; Lau et al.: 31.3 vs 30.9 kg/m^2^). BMI demonstrated variable associations with stiffness indices, including positive correlations with cfPWV (*r* = 0.24, *p* < 0.01), central pulse pressure (*r* = 0.33, *p* < 0.001), and AIx (*r* = 0.23, *p* = 0.01), but a negative correlation with CAVI (*r* = −0.20, p < 0.001). Importantly, sex differences in HFpEF remained significant after adjustment for BMI, indicating that adiposity contributes to but does not fully explain the observed disparities. Findings were narratively synthesised by combining studies by arterial stiffness index, and comparing the direction and consistency of sex differences between studies.

### 3.5 Measurement methods and heterogeneity

Measurement approaches were heterogeneous across included studies, as shown in Table 4. The most commonly utilized techniques included tonometry-based systems such as SphygmoCor for AIx, augmentation pressure (AP), and pulse wave velocity (PWV) (*n* = 3) [18], [23], [24]. One study utilized alternative device VaSera VS-1000 CAVI [25], while another used Mobil-O-Graph for central hemodynamics [26]. Echocardiography-based measures of Effective arterial elastance (Ea), end-systolic elastance (Ees), and ventricular-vascular coupling were reported in three studies: Gori et al., 2014, Beale et al., 2019 (TOPCAT), and Beale et al. (2019) (Invasive study) [19], [20], [21].

Adjustment for potential confounders varied widely across studies. Four studies controlled for demographic and clinical variables such as age, sex, BP, BMI, and comorbidities including hypertension, diabetes, and smoking [18], [22], [23], [24]. Three studies additionally adjusted for echocardiographic parameters (e.g., LV geometry, diastolic indices) and natriuretic peptides [19], [20], [21]. Two studies incorporated medication use into their models [23], [25]. In contrast, El Fol et al. (2022) did not specify any adjustment variables [26]. Disparity in method of arterial stiffness measurement, heterogeneity of the HF phenotype and inconsistent adjustment for confounders including BMI, comorbidities and medication use were major sources of heterogeneity.

## 4 Discussion

This review highlights important sex-related differences in arterial stiffness among individuals with HF and to our knowledge, represents the first systematic effort to synthesize evidence in this understudied area. Overall, the findings indicated that women exhibit greater vascular and pulsatile stiffness than men; however, the magnitude and consistency of these differences vary depending on the arterial index and the method of assessment. Across studies, sex differences were observed in both HFpEF and HFrEF cohorts, but were more consistent and more pronounced in HFpEF, where vascular stiffening plays a critical role in pathophysiology.

A key observation across multiple studies was that indices of wave reflection, especially AIx and AP were consistently higher in women. This pattern was evident across applanation tonometry, oscillometric systems, and invasive waveform analyses, and was observed in both HFpEF and HFrEF. All studies reporting AIx found higher values in women, even when absolute magnitudes varied. These findings suggest that women experience greater pulsatile load and earlier wave reflection, mechanisms that can contribute to higher LV filling pressures and impaired diastolic reserve.

In contrast, measures of arterial stiffness, such as cfPWV, showed less consistent sex differences, with several studies reporting no significant variation between men and women. Collectively, the inconsistent PWV findings and consistent differences in AIx/AP indicate that wave-reflection characteristics likely represent the more sensitive markers of sex-related vascular differences in HF.

Across studies assessing Ea as an integrated measure of vascular load, women consistently exhibited higher Ea [18], [19], [21]. Although some attenuation of differences occurred after indexing for body size in individual studies [19], the overall trend of higher pulsatile/arterial load in women persisted. This finding held true in echocardiography-derived Ea, invasive elastance measurements, and pressure waveform analyses.

Across all studies evaluating sex-specific vascular stiffness in HF, a coherent pattern emerged within HFpEF cohorts: women exhibited higher AIx, higher AP, higher Ea, and lower systemic or pulmonary compliance. Lau et al. (2022) demonstrated markedly higher AP and AIx in women with HFpEF, differences that remained after adjustment for clinical factors [18]. Similarly, Schott et al. (2023) identified significantly higher AIx in women within HFpEF [24], supporting the robustness of these findings across populations.

Studies evaluating ventricular–vascular coupling also demonstrated consistent sex-related differences. Women showed higher Ea, higher Ees, and higher diastolic stiffness (Ed), reflecting both vascular and myocardial contributions to increased load. These findings were particularly striking in invasive HFpEF cohorts, where women presented with higher filling pressures relative to workload (ΔPCWP/ΔCO) and lower systemic and pulmonary arterial compliance, a haemodynamic profile indicative of poor diastolic reserve. Although some echocardiographic estimates reported attenuation after body-size adjustment [19], the direction of effect remained consistent across studies.

Sex-specific differences extended beyond systemic circulation. Crosier et al. (2023) demonstrated that women with HFpEF had higher characteristic Zc and lower pulmonary arterial compliance [22], indicating more pronounced proximal aortic and pulmonary vascular stiffening. These findings complement invasive studies highlighting steeper workload-indexed rises in PCWP and greater exercise-induced pulsatile load in women [21]. Li et al. further reported slightly higher CAVI in women with chronic HF [25], again reinforcing the consistent directionality of greater stiffness in women.

Overall, these findings suggest that women with HFpEF exhibit a distinct hemodynamic phenotype of disproportionately increased wave reflection, higher pulsatile load, lower vascular compliance, and impaired ventricular–vascular coupling. Such differences likely contribute to higher filling pressures, impaired diastolic function, and reduced exercise tolerance, all of which are hallmarks of HFpEF.

Several mechanistic explanations support these sex-specific patterns. Women generally have smaller arterial diameters and higher heart rates, both of which amplify wave reflection and increase augmentation index [27], [28]. Importantly, these factors are well-established physiological determinants of wave reflection and may influence augmentation index independently of HF pathophysiology.

Hormonal influences, particularly the decline in estrogen after menopause, may accelerate vascular stiffening [29]. Together with comorbidities such as hypertension and obesity, these factors likely enhance pulsatile load and impair ventricular–vascular interaction [30]. The observed vascular abnormalities may reflect the burden of underlying comorbidities that promote endothelial dysfunction and vascular remodelling [31]. Among these, diabetes is particularly relevant, as it is associated with endothelial impairment, increased arterial stiffness, and adverse outcomes in HFpEF [32]. However, diabetes status was not consistently reported or adjusted for across the included studies, and its contribution to the observed sex differences cannot be fully excluded. Invasive and imaging studies consistently show poor diastolic reserve during exercise, further linking vascular pathophysiology to symptomatic limitations [18], [21].

Studies by Russo et al. (2012) emphasised the role of vascular stiffness and wave reflection in women's diastolic dysfunction, supporting our interpretation that increased wave reflection compromises ventricular–vascular coupling [33]. Pathophysiological studies further explain these differences through hormonal influences and vascular ageing [34], [35]. Although women in several cohorts had higher BMI, this measure is not a specific marker of adipose tissue mass and does not capture fat distribution or body composition [36]. While BMI showed variable associations with stiffness indices, the sex differences in HFpEF remained significant after adjustment for BMI, indicating that adiposity contributes to but does not fully explain these vascular disparities.

Importantly, hypertension, which is highly prevalent in HFpEF and a common comorbidity that increases vascular stiffness [37], was not uniformly adjusted for across studies. While several analyses included hypertensive status as a confounder, others did not, meaning that the influence of hypertension as a potential confounder cannot be fully excluded. This may introduce residual confounding, as hypertension may differ in prevalence or severity between sexes. Consequently, in studies that do not adequately adjust for hypertension, sex differences in arterial stiffness may partly reflect differences in hypertension burden as opposed to sex-related vascular characteristics.

These sex-specific vascular and haemodynamic differences have significant clinical implications. Higher arterial stiffness and wave reflection in women may exacerbate LV filling pressures, worsen diastolic reserve, and contribute to exercise intolerance. This underscores the need for sex-specific therapeutic approaches, including strategies aimed at reducing vascular stiffness, optimising blood pressure, and improving arterial compliance. Exercise-based rehabilitation is particularly promising, with studies demonstrating improvements in oxygen utilisation, exercise capacity, and diastolic function following structured training, benefits that may be especially relevant for women with HFpEF [38]. Furthermore, evaluation of wave reflection indices like AIx could provide further data on ventricular–vascular coupling and vascular stress that goes beyond traditional haemodynamic measurements. These indices may help identify higher-risk phenotypes and promote more personalised risk stratification in future clinical practice, even if they are not currently included in routine HF assessment.

Finally, heterogeneity in measurement techniques and adjustment for confounders across studies warrants caution in interpretation. Devices ranged from applanation tonometry (SphygmoCor) [18], [23], [24] to oscillometric systems (Mobil-O-Graph) [26] and echocardiographic calculations of Ea [19], [20], [21], while statistical models varied from basic demographic adjustments to comprehensive inclusion of cardiac structure, biomarkers, and medication use. However, despite methodological variability, the direction and consistency of findings, particularly for wave reflection and ventricular–vascular coupling parameters were remarkably stable across the literature.

### 4.1 Strengths

The present review used a comprehensive search strategy across databases and inclusion of both observational studies and randomised controlled trials. To our knowledge, this is the first review focusing specifically on sex-related differences in arterial stiffness in HF.

### 4.2 Limitations

Several limitations warrant consideration. Many of the included studies were constrained by small sample sizes and observational designs, limiting the ability to draw causal inferences. Although a structured, domain-based appraisal was undertaken, the use of an adapted checklist rather than a standardised risk-of-bias tool may limit direct comparability with other systematic reviews. In addition, substantial heterogeneity in arterial stiffness measurement techniques and incomplete BMI reporting prevented the conduct of a meta-analysis. Furthermore, although studies were grouped according to heart failure subtype (HFpEF, HFrEF, and mixed HF), no studies specifically evaluating individuals with Heart failure with mildly reduced ejection fraction (HFmEF) were identified, limiting the generalisability of the findings to this subgroup. The search strategy was limited to MEDLINE and CINAHL, and therefore relevant studies indexed in other databases such as Embase, Scopus, or the Cochrane Library may have been missed. Finally, the studies were limited to those published in English, or to non-English studies with available translations, possibly resulting in language bias. In addition, inconsistent adjustment for key confounders such as hypertension across studies may have introduced residual confounding, limiting the ability to isolate the independent effect of sex on arterial stiffness.

### 4.3 Future recommendations

Future research should prioritize the standardization of arterial stiffness measurement techniques, while ensuring consistent reporting of sex-stratified data and adiposity metrics. Equally important is the inclusion of adequately powered, sex-balanced cohorts to clarify mechanistic pathways linking vascular stiffness, adiposity, and HF phenotypes. In addition, randomised controlled clinical trials are needed to determine whether interventions specifically targeting arterial load and body composition can improve outcomes through sex-specific approaches.

## 5 Conclusion

In summary, this systematic literature review provides evidence that sex differences in arterial stiffness are more pronounced for wave reflection indices than for measures of aortic stiffness. While the available evidence remains limited, the findings highlight the importance of considering sex in the assessment and management of HF. Overall, the review emphasises the need for a personalised approach and further research is required to optimise care for both women and men with HF. These findings suggest that women may experience greater pulsatile load and impaired ventricular–vascular coupling, contributing to their higher symptom burden. While BMI correlated variably with stiffness indices, sex differences persisted after adjustment, indicating that adiposity contributes but does not fully explain the female-predominant phenotype.

## Human Ethics and Consent to Participate

Not applicable. This study is a systematic literature review that involved no human participants.

## CRediT authorship contribution statement

**Sara Younas:** Writing – original draft, Methodology, Formal analysis, Data curation. **Nduka C. Okwose:** Writing – review & editing, Supervision, Formal analysis, Data curation, Conceptualization. **Amy S. Fuller:** Writing – review & editing, Supervision, Formal analysis, Data curation. **Renae J. Stefanetti:** Writing – review & editing, Supervision, Formal analysis, Data curation. **Sarah J. Charman:** Writing – review & editing, Supervision, Conceptualization. **Fatima Bano:** Writing – review & editing. **Milan Dobric:** Writing – review & editing, Validation. **Milovan Bojic:** Writing – review & editing, Validation. **Petar M. Seferovic:** Writing – review & editing, Validation. **Prithwish Banerjee:** Writing – review & editing, Supervision, Project administration, Conceptualization. **Djordje G. Jakovljevic:** Writing – review & editing, Visualization, Validation, Supervision, Resources, Project administration, Conceptualization.

## Consent to Participate

Not applicable. No human participants were involved in this research.

## Funding

This systematic review was supported by the European Union's Horizon Europe research and innovation programme under grant agreement number 101080905 (STRATIFYHF) and by UK Research and Innovation under grant reference number 10073472.

## Declaration of competing interest

The authors declare that they have no known competing financial interests or personal relationships that could have appeared to influence the work reported in this paper.
