# Supplementary material for: Sex differences in vascular function among individuals with heart failure: a systematic review
Source: Int J Cardiol Heart Vasc. 2026 Jul 28;65:101979. doi: 10.1016/j.ijcha.2026.101979 (PMC13447576; doi:10.1016/j.ijcha.2026.101979)
Supplement: Supplementary file 1 [file mmc1.docx]

**Supplementary Material**

**Table 1. Study characteristics**

| Study | Country | Setting | Design | Sample Size (Total, M/F) | Age (M/F) | Type of HF | Main Finding |
| --- | --- | --- | --- | --- | --- | --- | --- |
| Crosier et al., 2023 | Canada | University of Ottawa Heart Institute | Observational (Cross-sectional) | 58 (19/39) | 78.3±7.1 / 74.1±7.4 | HFpEF | Women showed higher central BP and Zc, lower PAC |
| Beale et al., 2019  (TOPCAT) | Australia | Secondary analysis of TOPCAT trial (Americas cohort only) | Post-hoc RCT analysis | 654 (339/315) | 71(67–76)/73(69–78) | HFpEF | Women exhibited higher Ea, Ees, Ed |
| Gori et al., 2014 | USA/ multicentre | Secondary care | Phase 2 RCT | 279 (120/159) | 70±8 / 72±9 | HFpEF | Higher Ea in women compared with men. |
| Schott et al., 2023 | Germany | Community cohort (elderly population of Halle (Saale) | Cross-sectional | 767 (413/354) | NR | HFpEF | Women showed higher AIx |
| Lau et al., 2022 | USA | Tertiary care center | Observational study (Cross-sectional) | 190 (83/107) | 62±13 / 59±14 | HFpEF | Women shows higher AP, AIx |
| Beale et al., 2019  (Invasive) | Australia | Alfred Hospital, Melbourne (2008–2018) | Retrospective observational | 161 (47/114) | 68(63–74)/71(67–76) | HFpEF | Women higher PCWP/workload, higher Ea |
| El Fol et al., 2022 | Egypt | Cairo University Hospital | Observational (Prospective paired cohort) | 100 (80/20) | ~51.6±6.1  (sex-stratified age not reported) | HFrEF | Women higher AIx@75 and PP; PWV similar |
| Li et al., 2016 | China | Heart Failure Clinic of Zhejiang Hospital | Observational (Cross-sectional) | 354 (168/186) | 69.1±6.9 / 67.4±7.4 | Mixed HF | Women shows higher CAVI |
| Luers et al., 2017 | Germany | German Competence Network Heart Failure | Observational (Cross-sectional) | 257 (121/136) | ~66 overall | HFpEF/at risk | Women higher AIx@75; cfPWV similar |

AIx = Augmentation Index; AIx@75 = Augmentation index at 75 beats per minute; AP = Augmentation Pressure; AoPP = Aortic Pulse Pressure; CAVI = Cardio‑Ankle Vascular Index; cfPWV = Carotid‑Femoral Pulse Wave Velocity; Ea = Effective Arterial Elastance; Ed = Diastolic Stiffness; Ees = End‑Systolic Elastance; HF = Heart Failure; HFpEF = Heart Failure with Preserved Ejection Fraction; HFrEF = Heart Failure with Reduced Ejection Fraction; NR = Not reported; PAC = Pulmonary Arterial Compliance; PWV = Pulse Wave Velocity; SAC = Systemic Arterial Compliance; Zc = Impedance.

**Table 2.1 Quality appraisal for all non-randomised controlled trials**

|  | Beale et al. 2019 (TOPCAT) | Crosier et al. 2023 | El Fol et al. 2022 | Lau et al., 2022 | Li et al. 2016 | Luers et al., 2017 | Beale et al., 2019  (Invasive) | Schot et al. 2023 |
| --- | --- | --- | --- | --- | --- | --- | --- | --- |
| Representative Sample | Yes  TOPCAT trial sub‑cohort; multicentre). | Unclear  Not consecutive, single‑centre, n=58). | Unclear  Specific population, single‑centre, n=100). | Unclear  Study conducted at a single tertiary care center among referred HFpEF patients for invasive cardiopulmonary testing, which may limit representativeness. | Yes  Participants were recruited from a heart failure outpatient clinic. | Yes  Participants were drawn from the DIAST-CHF study, a large, multicentre cohort investigating diastolic dysfunction and heart failure in at-risk or diagnosed patients. | No  Patients recruited from a single tertiary hospital who underwent invasive RHC for clinical or research reasons | Yes  Derived from the population-based CARLA cohort (elderly residents of Halle, Germany), designed to represent the local community |
| Inclusion/exclusion criteria clearly defined | Yes  Described | Yes  Described | Yes  Described | Yes  Described | Yes  Described | Yes  Described | Yes  Described | Yes  Described |
| Are important confounders identified and adjusted for? | Yes  Adjusted for comorbidities, BNP and echocardiographic parameters. | Yes  Adjusted for age, BMI, HR, hypertension, diabetes, and smoking. | No  No multivariable adjustment. | Yes  Adjusted for age, BMI, hypertension, diabetes, smoking, prior MI, HR (for stiffness indices). | Yes  Multivariable regression adjusted for age, BMI, BNP, E/A ratio, ARB use, and FEV1; results adjusted for medication and comorbidities (hypertension, CHD). | Yes  Multivariate regression models adjusted for key confounders including age, sex, blood pressure, BMI, and cardiovascular risk factors/medications. Sex also entered as a covariate in models predicting LVFP. | Yes  Adjusted for body size and workload, but not all clinical confounders | Yes  Multivariable logistic and linear regression adjusted for age, sex, height, heart rate, mean arterial pressure, antihypertensive use, LVEF, AF, diabetes, lipids, creatinine, hsCRP. |
| Are the exposure / intervention and outcomes measured reliably and validly? | Yes | Yes | Yes | Yes | Yes | Yes | Yes | Yes |
| Were dropouts reported? How were missing values handled? | Unclear  Not detailed | Unclear  Not detailed | Yes  Re‑hospitalised excluded from follow‑up | Unclear  Not detailed | Yes  9 of 363 participants were excluded due to incomplete data | Unclear  Not detailed | Unclear  Not detailed | Yes  Participants with missing key variables were explicitly excluded from analysis |
| Were all planned outcomes reported? | Yes | Yes | Yes | Yes | Yes | Yes | Yes | Yes |
| Any funding, conflicts? | Unclear  No competing interests disclosed; no funding source reported. | Yes  Funding sources reported; Conflicts of interest declared. | Yes  Self-funded; No competing interests declared. | Yes  Funding acknowledged;  No conflicts | Yes  Funding acknowledge; No conflicts. | Yes  Funding source reported; no conflicts of interest declared. | Unclear  Conflicts of interest were declared, but no funding source was reported | Yes  Funded, no competing interest |
| Overall risk of bias | Low | Moderate | High | Moderate | Low | Low | Moderate | Low |

AF = Atrial Fibrillation; ARB = Angiotensin Receptor Blocker; BMI = Body Mass Index; BNP = B‑type Natriuretic Peptide; BP = Blood Pressure; CHD = Coronary Heart Disease; cfPWV = Carotid‑Femoral Pulse Wave Velocity; CAVI = Cardio‑Ankle Vascular Index; Ea = Effective Arterial Elastance; Ed = Diastolic Stiffness; Ees = End‑Systolic Elastance; FEV₁ = Forced Expiratory Volume in 1 Second; HF = Heart Failure; HFpEF = Heart Failure with Preserved Ejection Fraction; HFrEF = Heart Failure with Reduced Ejection Fraction; HR = Heart Rate; HTN = Hypertension; LVFP = Left Ventricular Filling Pressure; LVEF = Left Ventricular Ejection Fraction; MAP = Mean Arterial Pressure; NR = Not Reported; PAC = Proximal Aortic Compliance; PWV = Pulse Wave Velocity; SAC = Systemic Arterial Compliance; SVR = Systemic Vascular Resistance; Zc = Characteristic Impeda

**Table 2.2 Quality appraisal for RCT**

| Study | Gori et al., 2014 |
| --- | --- |
| Random sequence generation | Yes, phase 2, randomised, double blind, parallel-group, double blind, multicentre controlled trial |
| Allocation concealment | Yes |
| Blinding of participants | Yes |
| Blinding of outcome assessment | Yes |
| Incomplete outcome data | Yes |
| Selective reporting | Yes |
| Other (Funding + Conflict of interest | Yes |
| Overall risk of bias | Low risk |

**Table 3. Sex differences in arterial stiffness**

| Study ID | HF Type | Sex Difference Observed |
| --- | --- | --- |
| Fo et al. (2022) | HFrEF (decomp/comp) | Women had higher AIx@75 vs men (21.7% vs 14.1%) and pulse pressure; PWV similar between sexes |
| Beale et al. (2019) | HFpEF | Women had higher Ea vs men (2.4 vs 1.8 mmHg/mL; p <0.001), Ees (3.8 vs 2.7), and Ed (0.26 vs 0.19) |
| Crosier et al. (2023) | HFpEF | Women showed higher Zc (268.8 vs 216.7 dyn·s·cm⁵; p = 0.021) and lower PAC (4.3 vs 5.4 ×10⁶ cm⁴/dyne; p = 0.010); cfPWV slightly lower in women (p = 0.015) |
| Lüers et al. (2017) | HFpEF/risk | Women had higher AIx@75 (32% vs 24%, P<0.001); cfPWV did not differ significantly |
| Schott et al. (2023) | HFpEF | Women had higher AIx (33.6% vs 25.9%; p < 0.001); association of AIx with HFpEF stronger in men before adjustment |
| Beale et al. (2019) Invasive | HFpEF | Women had higher Ea (2 vs 1.5 mmHg/mL) and poorer diastolic reserve during exercise |
| Lau et al. (2022) | HFpEF | Women had higher AP (19.1 vs 13.7 mmHg; p = 0.003), AIx (28.9% vs 21.7%; p = 0.0002 , and AoPP (60.5 vs 56.7 mmHg; p <0.001); SEVR lower in women (p = 0.0001) |
| Gori et al. (2014) | HFpEF | Women had higher Ea (2.1 vs 1.8 mmHg/mL) and lower SAC (1.10 vs 1.31 mL/mmHg); higher LV stiffness |
| Li et al. (2016) | Mixed HF | Women had slightly higher CAVI (8.66 vs 8.47) |

AIx = Augmentation Index; AIx@75 = Augmentation Index normalized to a heart rate of 75 beats per minute; AP = Augmentation Pressure; AoPP = Aortic Pulse Pressure; BP = Blood Pressure; CAVI = Cardio‑Ankle Vascular Index; cfPWV = Carotid‑Femoral Pulse Wave Velocity; Ea = Effective Arterial Elastance; Ees = End‑Systolic Elastance; Ed = End‑Diastolic Elastance; HF = Heart Failure; HFpEF = Heart Failure with Preserved Ejection Fraction; HFrEF = Heart Failure with Reduced Ejection Fraction; LV = Left Ventricle; PAC = Pulmonary Arterial Compliance; PWV = Pulse Wave Velocity; SAC = Systemic Arterial Compliance; SEVR = Subendocardial Viability Ratio; Zc = Characteristic Impedance.

**Table 4: Measurement methods and heterogeneity**

| Study | Arterial stiffness measurement(s) | Device/ Technique | Confounders adjusted for |
| --- | --- | --- | --- |
| Lau et al., 2022 | AP, AIx, and AoPP (via radial artery tonometry) | SphygmoCor system | Age, BMI, HTN, diabetes, smoking status, and previous Myocardial Infarction. SphygmoCor derived measures (AP, Aix and AoPP were additionally adjusted for age and Heart rate. |
| Li et al., 2016 | CAVI | VaSera VS-1000 (Fukuda Denshi, Japan) | Age, BMI, Blood natriuretic peptides and E/A ratio. Multiple stepwise regression was adjusted for medication and history of hypertension and coronary heart disease. |
| Gori et al., 2014 | Ea | Blood pressure monitor, echocardiography | Ea indexed to height and Body surface area. Adjusted for age, diabetes, New York Heart Association (NYHA) class, history of Mycordial infarction, BMI, systolic blood pressure, presence of albuminuria and heart rate. |
| Beale et al., 2019 | pulse pressure, Ea, Ees, Ed, Ea/Ees | Blood pressure monitor, echocardiography | Age, sex, BMI, SBP, comorbidities (HTN, Diabetes), natriuretic peptides |
| El Fol et al., 2022 | cfPWV, AIx@75, AP, central PP | (Mobil-O-Graph 24-h device) | Not specified |
| Croiser et al., 2023 | cfPWV. Central BP, pulse pressure. Steady arterial load: SVR. Pulsatile arterial load: Zc), PAC, total arterial compliance, forward pressure wave amplitude, reflected pressure wave amplitude. Arterial wave reflection: AIx. | NIHem system (Cardiovascular Engineering, Norwood, Massachusetts),  Transthoracic Echocardiography | Age, BMI, Heart rate, HTN, diabetes, smoking |
| Luers et al., 2017 | cfPWV, Radial AIx@75 | SphygmoCor system (AtCor Medical, Sydney) | Age, gender, BP, medication use |
| Schott et al., 2023 | AIx | SphygmoCor | Age, sex, body height, diabetes, cholesterol, creatinine |
| Beale et al., 2019 | Ea | Standard cardiac catheterization laboratory setup with fluid-filled catheters for right and left heart pressures. And echo machine | Key hemodynamic indices indexed to body surface area and workload. |

AIx = Augmentation Index; AIx@75 = Augmentation index at 75 beats per minute; AP = Augmentation pressure; AoPP = Aortic pulse pressure; BMI = Body mass index; BP = Blood pressure; CAVI = Cardio‑ankle vascular index; cfPWV = Carotid‑femoral pulse wave velocity; Ea = Effective arterial elastance; Ed = Diastolic stiffness; Ees = End systolic elastance; HTN = Hypertension; PAC = Proximal aortic compliance; PWV = Pulse wave velocity; SAC = Systemic arterial compliance; SVR = Systemic vascular resistance; Zc = Impedance.
